# DNIC in whiplash and ankle-injured controls. 1-year prospective findings

**DOI:** 10.1186/1129-2377-14-S1-P179

**Published:** 2013-02-21

**Authors:** H Kasch, TS Jensen

**Affiliations:** 1 Danish Pain Research Center, Denmark

## Aim of investigation

The role of central processing of pain in the development of chronic neck pain and headache and work disablement after whiplash injury is not well understood. Studies have supported findings of spread of pain in chronified patients. This study examines development of masseter muscle tolerance to pressure pain during cold pressor counter-stimulation in whiplash and ankle-injured controls.

## Methods

Consecutively 141 acute WLP and 40 ankle injured recruited from emergency units were examined after 1 week, 1, 3, 6, 12 months obtaining neck/head VAS score, number-of-non-painful complaints, epidemiological, social, psychological data and neurological examination, active neck mobility, and furthermore muscle tenderness and pain response, strength and duration of neck muscles. Pressure pain tolerance threshold of masseter muscle was performed before and during the counter-stimulation by "The Cold Pressor Test" after 1, 3, 6 months. Based on initial findings within 1-week after injury risk factors derived (reduced CROM, intense neckpain/headache, multiple non-pain complaints were applied in a Risk Assessment Score and divided into 7 risk-strata.

## Results

Significant differences was found in strata after 1 month, but not after 3 and 6 months. Furthermore, after 3, 6 months all whiplash patients did not process pressure pain different from ankle injured controls during counter-stimulation.

## Conclusions

Change in central processing of pain by means of central inhibitory or facilitatory mechanisms may play a role in delayed recovery or even development of chronic disability after whiplash. But data presented here are only partly supportive of this.
